# Supplementary material for: Immobilization of Radioiodine via an Interzeolite Transformation to Iodosodalite
Source: Nanomaterials (Basel). 2020 Oct 29;10(11):2157. doi: 10.3390/nano10112157 (PMC7693607; doi:10.3390/nano10112157)
Supplement: Supplementary file 1 [file nanomaterials-10-02157-s001.pdf]

Supplementary Materials

Immobilization of Radioiodine via an Interzeolite Transformation to Iodosodalite

Hyejin An [1], Sungjoon Kweon [1], Sanggil Park [2], Jaeyoung Lee [3], Hyung-Ki Min [4,*] and Min Bum Park [1,*]

[1] Innovation Center for Chemical Engineering, Department of Energy and Chemical Engineering, Incheon National University, Incheon 22012, Korea; hjan_95@inu.ac.kr (H.A.); sjkweon@inu.ac.kr (S.K.)
[2] Nuclear Energy Team, Lee & Ko, Seoul 04532, Korea; sanggil.park@leeko.com
[3] School of Mechanical and Control Engineering, Handong Global University, Pohang 37554, Korea; jylee7@handong.edu
[4] LOTTE Chemical Research Institute, Daejeon 34110, Korea
* Correspondence: pulcherrima7@gmail.com (H.-K.M.); mbpark@inu.ac.kr (M.B.P.)

Table S1. Solubility products (Ksp) of the representative metal iodide compounds.

Compound.	Ksp at 25 °C
AgI	8.5×10^{-17}
CuI	1.1×10^{-12}
PbI_2	7.1×10^{-9}
TlI	6.5×10^{-8}
NaI	151

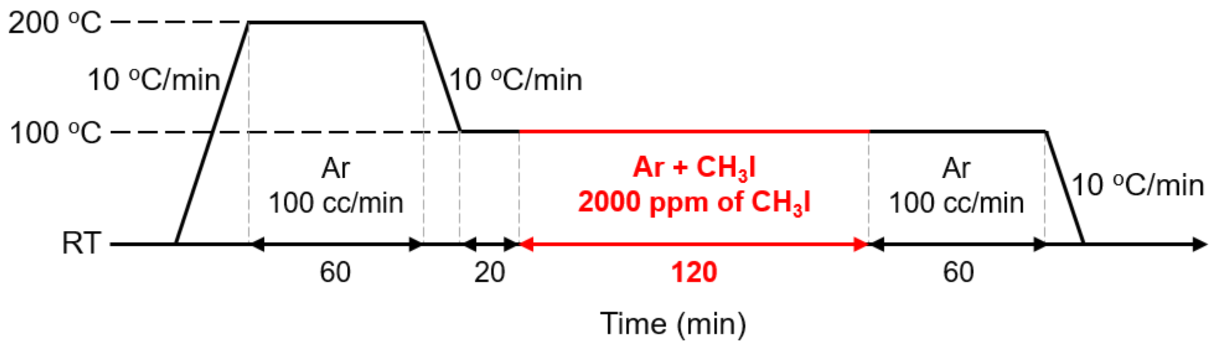

Figure S1. Schematic diagram of the treatments of Na- or Ag-zeolites for the adsorption and desorption of CH_3I.

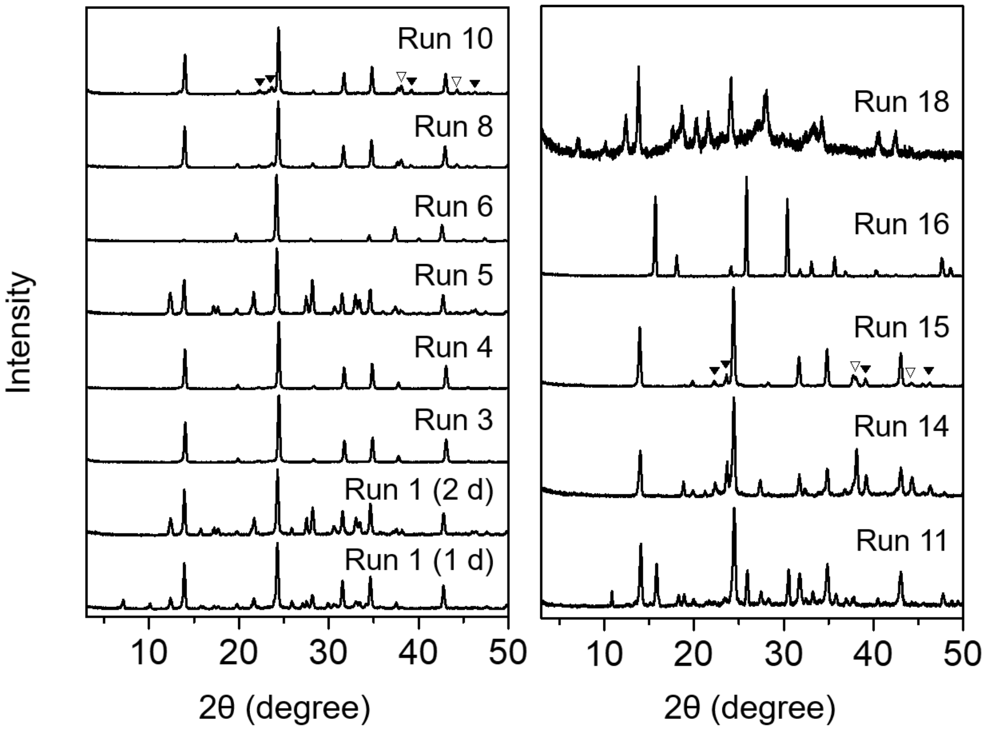

Figure S2. Powder XRD patterns of the products synthesized by the interzeolite transformation of Na-A and Na-X. The X-ray peaks from AgI and Ag metal are marked by closed (▼) and open inverted triangle (▽), respectively.

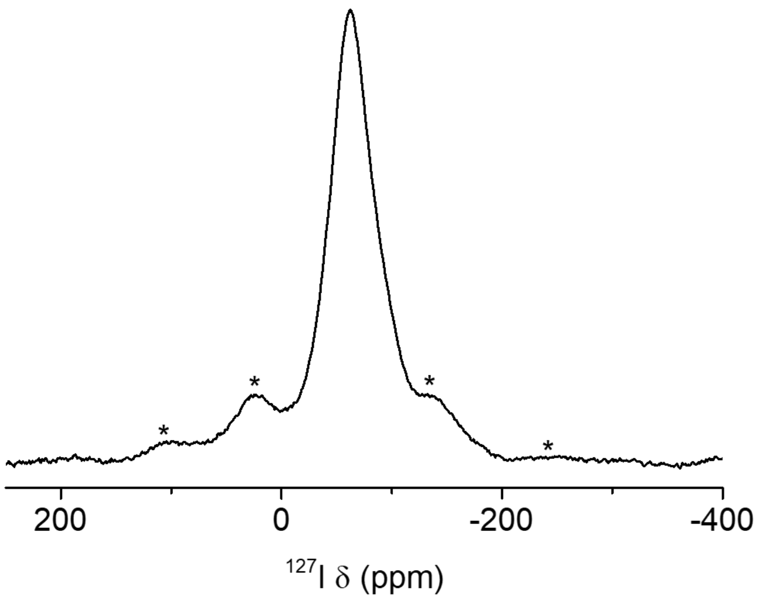

Figure S3. ^{127}I MAS NMR spectrum of AgI powder. The spinning side bands are indicated by asterisks.

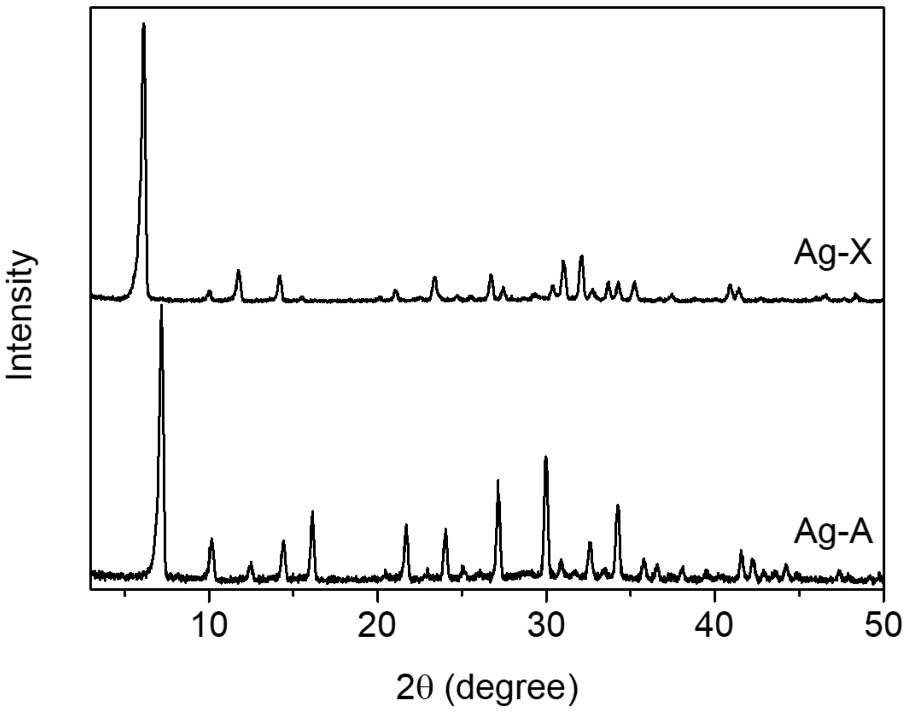

Figure S4. Powder XRD patterns of Ag-A and Ag-X zeolites.

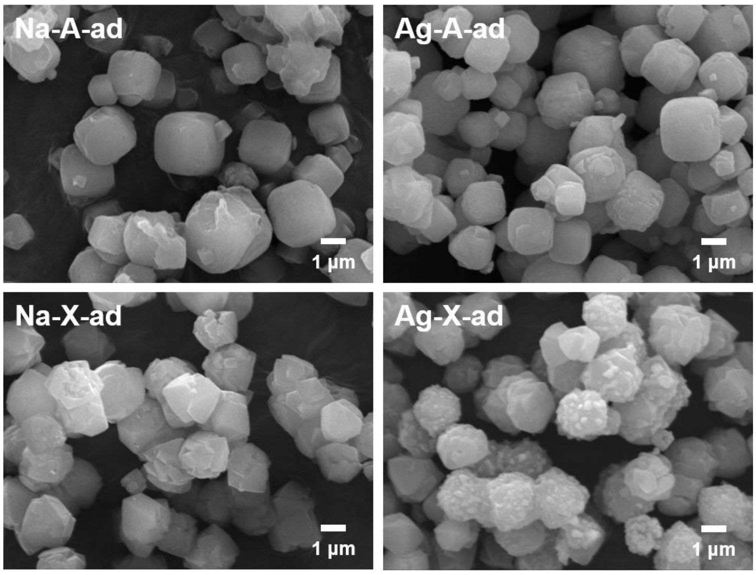

Figure S5. SEM images of the CH_3I adsorbed Na-A, Ag-A, Na-X, and Ag-X zeolites.
